# Cellulose Triacetate-Based Mixed-Matrix Membranes with MXene 2D Filler—CO_2_/CH_4_ Separation Performance and Comparison with TiO_2_-Based 1D and 0D Fillers

**DOI:** 10.3390/membranes12100917

**Published:** 2022-09-22

**Authors:** Chhabilal Regmi, Jalal Azadmanjiri, Vipin Mishra, Zdeněk Sofer, Saeed Ashtiani, Karel Friess

**Affiliations:** 1 Department of Physical Chemistry, University of Chemistry and Technology, Technická 5, 16628 Prague 6, Czech Republic; 2 Department of Chemical Engineering, University of Arkansas, Fayetteville, AR 72701, USA; 3 Department of Inorganic Chemistry, University of Chemistry and Technology, Technická 5, 16628 Prague 6, Czech Republic; 4 Department of Glass and Ceramics, University of Chemistry and Technology, Technická 5, 16628 Prague 6, Czech Republic

**Keywords:** cellulose triacetate, mixed-matrix membrane, MXene, TiO_2_ nanoparticles, TiO_2_ nanotube, gas separation

## Abstract

Mixed-matrix membranes (MMMs) possess the unique properties and inherent characteristics of their component polymer and inorganic fillers, or other possible types of additives. However, the successful fabrication of compact and defect-free MMMs with a homogeneous filler distribution poses a major challenge, due to poor filler/polymer compatibility. In this study, we use two-dimensional multi-layered Ti_3_C_2_T_x_ MXene nanofillers to improve the compatibility and CO_2_/CH_4_ separation performance of cellulose triacetate (CTA)-based MMMs. CTA-based MMMs with TiO_2_-based 1D (nanotubes) and 0D (nanofillers) additives were also fabricated and tested for comparison. The high thermal stability, compact homogeneous structure, and stable long-term CO_2_/CH_4_ separation performance of the CTA-2D samples suggest the potential application of the membrane in bio/natural gas separation. The best results were obtained for the CTA-2D sample with a loading of 3 wt.%, which exhibited a 5-fold increase in CO_2_ permeability and 2-fold increase in CO_2_/CH_4_ selectivity, compared with the pristine CTA membrane, approaching the state-of-the-art Robeson 2008 upper bound. The dimensional (shape) effect on separation performance was determined as 2D > 1D > 0D. The use of lamellar stacked MXene with abundant surface-terminating groups not only prevents the aggregation of particles but also enhances the CO_2_ adsorption properties and provides additional transport channels, resulting in improved CO_2_ permeability and CO_2_/CH_4_ selectivity.

## 1. Introduction

Membrane-based gas separation has been recognized as a feasible and effective technology at the laboratory and industrial scales. Likewise, it has attracted eminent attention as the major paradigm for separation [1]. Application of this technology demonstrates it is competitive with and can complement traditional separation techniques due to its compounded properties, which include a rapid mass transfer rate, a specific gas-selective nature, lower power consumption rate, cheapness, and environmental benignity, among others [2]. However, in assessing the performance of polymers as membranes, a strong antithetical relationship between permeability and selectivity is persistently encountered [3]. The selectivity–permeability trade-off—a known bottleneck of polymeric membranes in the gas separation process—has been resolved through the development of MMMs [4]. An MMM utilizes the advantages of both polymeric and inorganic materials, thus potentially providing improved performance without significantly increasing the fabrication cost [5].

Active research into the design and synthesis of new materials via incorporation of inorganic nanoparticles has been extensively carried out in recent years. One of the latest approaches includes the inclusion of such particles into polymeric membranes to increase membrane performances such as permeability, selectivity, strength, and hydrophilicity [6,7,8]. Thus, the advancement in MMMs subsequently depends, in turn, on the development of suitable inorganic filler materials [9]. A vast number of nanomaterials, such as zeolites, metal–organic frameworks (MOF), carbon nanotubes, graphene sheets, activated carbon, carbon molecular sieves, and metal oxides [10,11] have been widely incorporated into a polymeric matrix to fabricate MMMs. These filler particles possess different sizes and shapes, most commonly 0D, 1D, and 2D. Such materials offer a promising strategy for the construction of membranes with a tailored pore structure and well-defined pore size distribution, substantially favoring elevated permeability and selectivity [1]. Although every preparation uses inorganic fillers, the resulting morphology and membrane separation performance can vary significantly, primarily due to the different capabilities of these molecular sieves—either based on shape or size—in discriminating various components available in the feed mixture [6]. A difference in the structure and characteristics of fillers may eventually give rise to a difference in the separation efficiency of the resulting MMMs. Similarly, the inclusion of filler particles can also result in alterations to the properties of the neighboring polymer phase. In contrast, polymers can increase the filler density, which ultimately affects the overall membrane separation performance [12].

Notwithstanding the benefits of the MMM-based separation process, many challenges still need to be confronted to enable the large-scale industrial implementation of such membranes [13]. A key challenge is characterizing the transport of species across the membrane and its dependence on inherent filler–matrix properties. Among others, 2D inorganic materials have evoked predominant attention in gas separation processes. These fillers possess sheet-like structures with atomic-level size and thickness, allowing them to be utilized in constructing transport channels of resultant gas separation membranes [14,15]. Similarly, the barrier created by such layered materials allows for discriminatory transfer of different gases through the membrane based on size sieving and/or electrostatic repulsion. Furthermore, their ultra-thin thickness can facilitate the transport of molecules or ions while perpetuating a preferable blocking effect. Thus, the design of nanochannels with 2D molecular sieve materials has aroused considerable interest and efforts [1]. The sheet-like fillers are either randomly distributed or align themselves (more or less) parallel to the membrane surface after their incorporation into the polymer matrix. This arrangement ultimately distorts the diffusion paths, thus increasing membrane selectivity by creating relatively higher resistance to larger molecules [4,16]. Similarly, these post-formed channels also lead to a gas barrier effect, counteracting the increase in gas permeability [17]. Instead, a pre-structured material such as MXene, which possesses intrinsically uniform effective channels, prevents uncontrollable packing during membrane preparation, thereby potentially and directly affecting the membrane separation performance [18]. The versatile chemistry of MXenes, a 2D layered material, has already been applied in different applications, including energy storage, biosensors, lubricating agents, and catalysis [1]. Therefore, the coupling of membrane separation technology with MXene is an emerging area of application. Although graphene oxide [19] and MoS_2_ [20] have attracted plenty of interest in the membrane community, one of the essential advantages of MXenes, compared with these oxides, is their higher compatibility with the polymer matrix [21].

Similarly, layered atomic structures constructed on covalent bonding and evenly distributed functional groups such as –OH, –O–, and –F impart MXene with excellent mechanical rigidity and thermal stability, and they have shown great promise as nanofillers in polymer-based membranes for gas separation [22]. Thus, the rational design of MXene-based membranes has dramatically improved over the last few years. Many studies have assessed MXene-based MMMs for water purification and pervaporation compared with other 2D fillers; however, the use of MXene as a filler in natural/biogas separation applications is still in the infancy stage. Luo et al. have synthesized MXene/PEG MMMs and revealed an ideal selectivity of 32.18 for CO_2_/N_2_ and 27.84 for the CO_2_/CH_4_ gases pair [23]. Guan et al. have used pre-structured MXene/Pebax MMMs and demonstrated a CO_2_/N_2_ selectivity of 104.85 [18]. Similarly, Liu et al. have synthesized a Pebax-based membrane filled with 2D MXene nanosheets and displayed a separation performance with a CO_2_ permeance of 21.6 GPU and a CO_2_/N_2_ selectivity of 72.5 [24]. However, to the best of our knowledge, there are no reports on the use of MXene nanosheets as a filler to prepare cellulose triacetate based MMMs for gas separation.

Generally, the most critical factor influencing the success of MMMs is sufficient compatibility between the continuous (polymers) and disperse (filler) phases. Aside from adjusting the surface chemical configuration of the fillers to reduce the gaps between filler and polymer, proper adjustment of the shape and size of particles has also been demonstrated as an efficacious approach to circumventing nonselective defect formation and improving the gas permeation efficiency [25].

Several studies have been published on the effect of filler size on gas separation efficiency. Tantekin-Ersolmaz et al. discovered a decrease in gas permeability with larger-sized filler particles as compared to the fillers with relatively smaller particle size. Silicate-PDMS MMMs loading with different sizes of fillers were compared in the study. The observed results were presumed likely due to the large area and abundant filler–polymer interfaces that the gas molecules have to cross in these cases [26]. Coronas et al. have stated that, compared with nanosized particles (MCM-41), micron-size particles (MCM-48) could provide a lower external surface area and reduce aggregation, and as a consequence improve the dispersibility and interaction with the polymer and, thus, providing higher efficiency [27]. However, these two abovementioned results contradict each other. In another study, Hashemifaed et al. used halloysite nanotubes (HNTs) with large pore size as fillers and reported on different interfacial phases for MMMs, comprising five representative morphologies (i.e., ideal, void, rigidified, pore-blocking, and agglomeration combined with pore-blocking). After comparison with minor pore size fillers [28], they concluded that the type of filler (large or small pore size), as well as the filler particle size, was among the most influential factors determining the separation efficiency/performance of MMMs [29].

Meanwhile, only a handful of studies have focused on the effect of filler shape on the gas separation behavior of MMMs. Kaliaguine et al. have emphasized that the permeability and selectivity of various polymer matrixes for the separation of different gas pairs can also be improved by the shape selectivity and specific adsorption properties of zeolitic crystals, combined with the fluent processibility of the polymer matrix [30]. Suen et al. have compared the gas selectivity of polyether sulfone (PES)-based MMMs containing two different fillers with different shapes/sizes. They compared the efficiency of a lamellar Na-montmorillonite clay (mean length of 500 nm) and spherical TiO_2_ particles (70 nm) and concluded that the spherical TiO_2_ particles formed an ideal interface morphology, thus obtaining better gas separation performance than MMMs containing lamellar MMT sheets [31].

In this study, we first explore the effect of different MXene filler loadings on the physicochemical properties and, hence, gas separation performance of cellulose triacetate-based membranes. The gas separation efficiency of this 2D filler is simultaneously compared with that of 1D and 0D fillers to reveal guiding principles for future work in this field.

## 2. Materials and Methodology

### 2.1. Chemicals

Titanium dioxide (Degussa, 99.9% purity, Sigma-Aldrich, St. Louis, MI, USA), titanium dioxide nanoparticles (>99.5%, Sigma-Aldrich), Ti_3_AlC_2_ MAX phase material (Jinzhou Haixin Metal Materials, China), hydrofluoric acid (HF, Sigma-Aldrich), cellulose triacetate (CTA, acetyl content 43–44%, Acros Organics), 1-methyl-2-pyrrolidinone (NMP, ACS reagent >99.0%, Sigma-Aldrich), sodium hydroxide (NaOH, 98.99% purity, Duksan Chemical), and Hydrochloric acid (HCL, ACS reagent >99.0%, Duksan Chemical) were mainly used for the synthesis of fillers as well as membranes. All chemicals were used as received without any further purification. Similarly, single gases (CO_2_, CH_4_, C_3_H_8_, C_3_H_6_, and SF_6_) with a purity of >99.95% (Linde Gas) were used as received.

### 2.2. Synthesis of Fillers Ti_3_C_2_T_x_ MXene, TiO_2_ Nanotubes, and TiO_2_ Nanoparticles

Multi-layered Ti_3_C_2_T_x_ MXene was synthesized following the method previously reported by Chia et al. [32]. Briefly, 5.0 g of Ti_3_AlC_2_ MAX phase material was mixed with 250 mL HF (40 wt.%) solution and continuously stirred under at room temperature for 7 days. Then, the sample was separated through repeated centrifugation and re-dispersion in water until a neutral pH value for the residual water was obtained. Subsequently, the sample was dried for 48 h in a vacuum oven set at 50 °C. Similarly, the TiO_2_ nanotubes were synthesized through a similar procedure, as explained in a previous work by Regmi et al. [33]. Commercially available TiO_2_ nanoparticles were used without any further modification.

### 2.3. Synthesis of MMMs

The MXene filler-based CTA MMM was prepared using the protocol of Regmi et al. [33]. Membranes with different concentrations of MXene fillers (1–5 wt.%) were prepared. In brief, firstly the calculated amount of MXene filler was mixed with NMP solvent (25 mL). The mixture was then sonicated for 30 min, and further stirred for 4 h. Then, 1.62 g of CTA polymer was added to the mixture and stirred overnight (24 h), maintaining an oil bath temperature of 70 °C. The mixture was then left undisturbed for about 4 h. The membrane was cast into a glass plate using an applicator (Elcometer 3580, Germany). The casted membrane was left in an ambient condition for 48 h. Further, the membrane was dried in a vacuum oven at 50 °C for 6 h to ensure complete evaporation of the solvent. Prepared membranes were labeled CM1, CM2, CM3, CM4, and CM5 for 1–5 wt.% filler concentration, respectively. An analogous protocol was used to synthesize other MMMs containing 0D and 1D fillers, and the respective samples are denoted by S-0D and S-1D throughout the manuscript.

### 2.4. Characterization

Fourier transform infrared spectroscopy (FTIR) spectra of the samples were recorded using an iS50R FTIR spectrometer (Thermo Scientific, USA) in the range of 4000–500 cm^−1^. Sixteen scans were accumulated with a resolution of 4 cm^−1^ for each spectrum in absorption mode. The morphology of the MXene was investigated using scanning electron microscopy (SEM) with an FEG electron source (Tescan Lyra dual-beam microscope). Membrane morphology and elemental distribution were analyzed using SEM/TOF-SIMS (scanning electron microscopy/time-of-flight secondary ion mass spectroscopy; Tescan, Czech Republic). The primary ion source was a Ga liquid metal ion gun. The primary ion source beam energy used was 30 keV, with a current of nearly 10 nA for surface abrasion. All the membrane samples were coated with gold (Au) for surface conductivity. Scanning electron microscopy (SEM, Tescan Lyra, Czech Republic; 15 kV accelerating voltage, SE detector) was used to evaluate the cross-section morphology of prepared samples possessing 0D, 1D, and 2D fillers. This microscopy was connected to energy-dispersive spectroscopy (EDS, Oxford Aztec, 80 mm^2^, High Wycombe, United Kingdom) and was further used to analyze the distribution of fillers in the polymer matrix (especially Ti). Prior to analysis, membranes were first dipped into liquid nitrogen, fractured into small pieces, and coated with Au using sputter coating. Transmission electron microscopy (TEM, JEM-2200FS, JEOL, Japan) was used to image the morphology of the nanofillers. The images were taken at an accelerating voltage of 200 kV in TEM imaging mode. The static contact angle values of the as-synthesized membranes were measured using a fully automated optical tensiometer (Attension Theta Flex Auto 3, Biolin Scientific) at ambient conditions. The contact angles of the membranes were measured using the sessile drop method. The camera captured the drop profile image of water, and a fitting method was used to determine the value of the contact angle. Each membrane was measured four times, and the maximum error was calculated as ±6°. Surface topography analysis of the synthesized membranes was carried out using a 3D optical profilometer (NewView 9000, ZYGO, USA). X-ray diffraction (XRD) measurements were carried out on a second-generation D2 Phaser X-ray diffractometer (Bruker, Germany) with Cu-Kα radiation (λ = 1.540 Å), Selected Bragg 2θ angular regions were measured from 5 to 80° in 0.02° increments at room temperature. Survey and high-resolution X-ray photoelectron spectroscopy (XPS) analyses of MXene nanofillers were conducted on ESCAProbeP Spectrometer (Omicron Nanotechnology Ltd., Germany), and the primary X-ray beam was monochrome radiation of an Al-Kα source (1486.7 eV). Thermal stability was determined by thermogravimetric analysis (TGA; TG5500, TA instrument, USA) in an N_2_ atmosphere (flow rate of 35 mLmin^−1^) from 25 to 800 °C at a scan rate of 10 °C min^−1^. Differential scanning calorimetry (DSC) (DSC3+, Mettler Toledo, Switzerland) was used for thermal analysis of the synthesized membranes. N_2_ was used as a purge gas, with a flow rate of 30 mL min^−1^. Each sample was placed in an aluminum crucible, and scanning was performed with a temperature rate of 10 °C min^−1^ from 25 to 400 °C.

### 2.5. Gas Sorption and Permeation Measurements

Gas sorption experiments were performed gravimetrically at 25 °C in a pressure range from 0.01 to 1.5 MPa, using a self-developed sorption apparatus equipped with a calibrator (McBain quartz spiral balance) [34]. An in-house designed sorption apparatus equipped with a calibrator (McBain quartz spiral balance) that gravimetrically evaluated the gas sorption efficiency of the prepared membrane materials was used. The absorption experiment was conducted at 25 °C in a pressure range from 0.01 to 1.5 MPa. Similarly, the gas permeation efficiency was determined using a tailor-made time lag permeation setup [35]. The experimental conditions and protocol for sorption and permeation were adapted from the previous work of Regmi et al. [33,36]. Membrane discs with an effective surface area of 2.14 cm^2^ were placed in the membrane permeation cell. Before starting the gas permeation experiment, all the trapped air inside the cell was evacuated using a vacuum at both ends. In addition, the permeation cell was regularly evacuated with a vacuum pump to remove the trapped gases inside the cell prior to each gas sorption experiment. The permeation data were recorded using the SWeTr version 1.13 (2003 Neovision, Czech Republic) software until the steady-state permeation was reached. The pressures on both the feed and permeated sides were maintained at 1.5 bar. Three consecutive measurements were taken for every gas studied, and the average value was calculated. The maximum experimental error was estimated to be 8–10%.

The permeability (*P_i_*; 1 Barrer = 10^−10^ cm^3^ (STP) cm cm^−2^ s^−1^ cmHg^−1^) of a gas *i* is given by

(1) $$P_{i}=D_{i}S_{i}$$

where *D_i_* and *S_i_* represent the diffusion and solubility coefficients of component *i*, respectively. Permeability was calculated by differentiating the pressure increase as a function of time, using the following equation:

(2) $$P=\frac{\;V_{d}l}{P_{2}ART}[\left(\frac{dp_{1}}{dt}\right)ss-\left(\frac{dp_{1}}{dt}\right)leak\;]$$

where $V_{d}$ is a downstream volume (cm^3^), *l* is the membrane thickness (cm), $P_{2}$ is the upstream absolute pressure (cmHg), *A* is the active surface area of the membrane (cm^2^), *T* is the temperature (K), *R* is the universal gas constant (cm^3^ cmHgmol^−1^ K^−1^), $\left(\frac{dp_{1}}{dt}\right)ss$ is the rate of downstream pressure rise during testing (cmHgs^−1^), and $\left(\frac{dp_{1}}{dt}\right)leak$ is the rate of downstream pressure rise under a vacuum (cmHgs^−1^).

The fluxes in the membrane module were kept constant during the experiments. The downstream pressure was raised linearly as a function of time, until a steady state was reached. This time duration, the so-called time lag (θ), was used for the evaluation of the diffusion coefficient (*D*) using the following equation:

(3) $$D=\frac{l^{2}}{6\theta }$$

where *l* is the membrane thickness.

The ideal selectivity was calculated as a ratio of permeabilities of a pair of pure gases (*x* and *y*) according to the following equation:

(4) $$\alpha_{xy}=\frac{P_{x}}{P_{y}}$$

## 3. Results and Discussion

**Figure 1:**
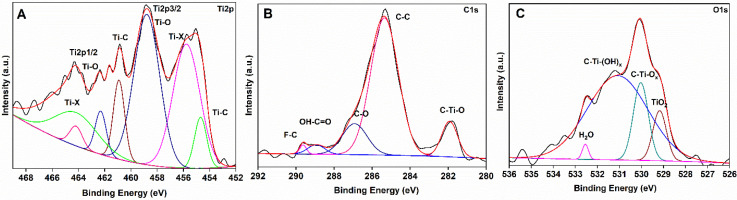
High resolution XPS spectra of; (**A**) Ti2p, (**B**) C1s and, (**C**) O1s for Ti_3_C_2_T_x_ MXene nanofiller.

Among different MXene materials, Ti_3_C_2_T_x_ was taken as filler in the CTA polymer matrix to facilitate selective gas transport. XPS survey spectra of MXene (Figure S1, Supplementary Materials) revealed the presence of Ti, C, O, and F. The high-resolution XPS spectra for Ti, C, and O for Ti_3_C_2_T_x_ MXene and their respective peak fits are displayed in Figure 1. Ti2p peaks centered at 454.7 and 461.6 eV suggest the formation of Ti–C bonds [37], and the calculated ∆BE of 6.9 eV between the Ti2p_3/2_ (455.7 eV) and Ti2p_1/2_ (462.3 eV) indicate the formation of Ti^3+^ species [38]. The C1s region deconvolutes into five different peaks at binding energies of 281.7, 285.3, 286.9, 288.9, and 289.8 eV. These peaks are assigned to Ti–C, C–C, C–O, OH–C=O, and C–F bonds, respectively [39], Similarly, in O1s, the binding energies at 529.1, 530.0, 531.0, and 532.5 eV are related to O–Ti, C–Ti–O, C–Ti–(OH)_x_, and H–O–H bonds, respectively [40].

**Figure 2:**
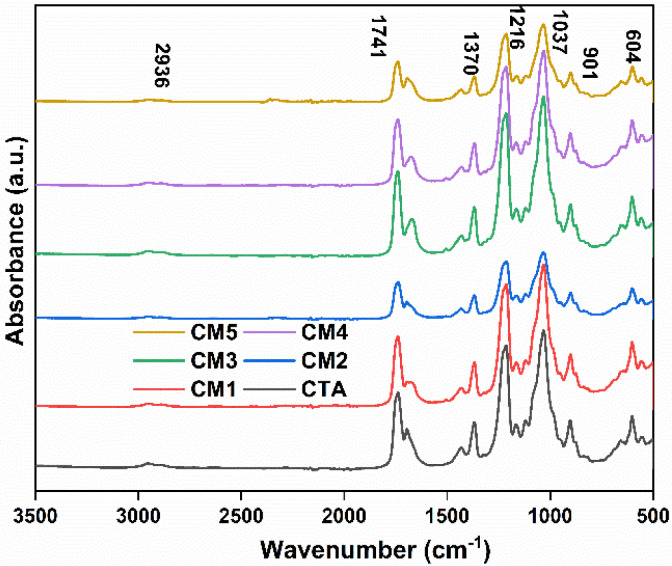
FTIR spectra of the synthesized MMM samples.

Figure 2 shows the FTIR spectrum of pristine CTA membrane and MXene-incorporated CTA-based MMMs. The absorption band located around 1741 cm^−1^ can be attributed to the stretching vibration of the C=O group. The absorption band at 1370 cm^−1^ represents the CH_3_ acetyl group. Bands at 1216 and 1037 cm^−1^ correspond to the stretching mode of C–O single bonds. The less intense band at 2936 cm^−1^ is ascribed to the C–H bond [41,42]. Figure S2 represents the FTIR spectra of the pristine MXene nanosheet. The absorption peak at 584 cm^−1^, which corresponds to the –OH group, is evidence of typical –OH vibration [43]. Similarly, the absorption peak centered at 650 cm^−1^ is assigned to the Ti–O group [38]. The absorption band at 3725 cm^−1^ is due to the –OH functional group out-of-plane vibration of MXene [44]. No significant difference in FTIR spectra between pristine CTA and corresponding MMMs was observed, except for a decrease in the characteristic peak intensity of CTA in MMMs with an increase in the concentration of loaded MXene nanofillers. This behavior can be ascribed to the decreased polymer concentration due to the presence of fillers, as well as the interactions of the polymer matrix with the MXene [45]. Figure S3 presents the XRD spectra of as-prepared MMMs, the MXene nanoparticles, and pristine CTA membrane. The broad peak of pristine CTA that centered at 19.5 2θ degrees depict the semi-crystalline nature of the polymer. The appearance of diffraction peaks of both fillers and polymer in the diffraction patterns of MMMs confirms the homogeneous distribution of MXene filler in the CTA matrix. Furthermore, the intensity of the MXene crystalline peaks intensifies with increased MXene loading concentration in MMMs, suggesting that the crystalline structure of MXene remains unchanged after being incorporated into the CTA matrix. Meanwhile, the broad peak of the polymer became relatively less noticeable.

**Figure 3:**
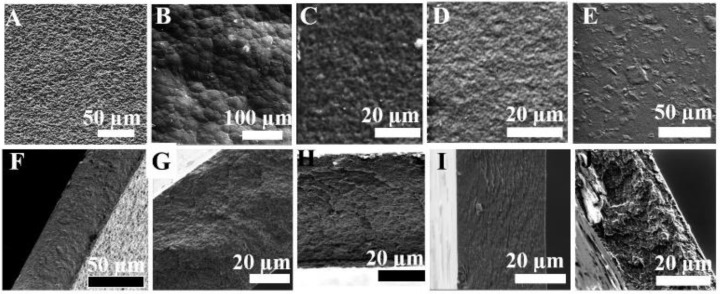
Surface and its corresponding cross-sectional morphologies of the prepared MMMs: (**A**,**F**) CM1; (**B**,**G**) CM2; (**C**,**H**) CM3; (**D**,**I**) CM4; and (**E**,**J**) CM5.

The surface properties of the fabricated membranes were found to be dependent on the amount of filler added. Figure 3 shows the surface morphology and cross-sections of the synthesized MMMs. The surface of each sample presents a fairly compact morphology, with ridges and valleys, and is free of defects such as pores and cracks. Cross-section images display a homogeneous phase, with solid and dense structure with defect-free morphology for all the synthesized membranes. No significant observable voids were seen at lower concentrations of the fillers. This indicates the excellent interfacial adhesion of the MXene surface with the CTA matrix. This further suggests the formation of hydrogen bonds between them. However, at a higher concentration, the aggregation of MXene results in increased sizes of void spaces between the polymer matrix and fillers, thus improving CO_2_ and CH_4_ permeability, which leads to deterioration of the gas separation selectivity.

Furthermore, the topography of the prepared membranes was studied using a 3D profilometer, as shown in Figure S4. The surface roughness of the membranes increases with the filler concentration. The roughness follows the order of CM1 (Sq, 0.187 µm) < CM2 (Sq, 0.381 µm) < (Sq, 0.468 µm) < CM4 (Sq, 0.476 µm) < CM5 (Sq, 0.517 µm). It is presumed that, as the filler concentration increased, an increased amount of nanofillers bonded to the membrane surface increased, leading to high roughness. The water contact angle of the synthesized membranes was also evaluated, and the results are displayed in Figure S5. A contact angle of 53.3° was reported for pristine CTA membrane in previous work [33]. The contact angles of all the synthesized MMMs were higher than that of the pristine membrane. The measured contact angle values for the synthesized samples are as follows: CM1, 60.98°; CM2, 64.82°; CM3, 65.17°; CM4, 71.82°; and CM5, 69.48°. With an increase in MXene loading, the water contact angle increases, indicating that the surface roughness is also increased.

**Figure 4:**
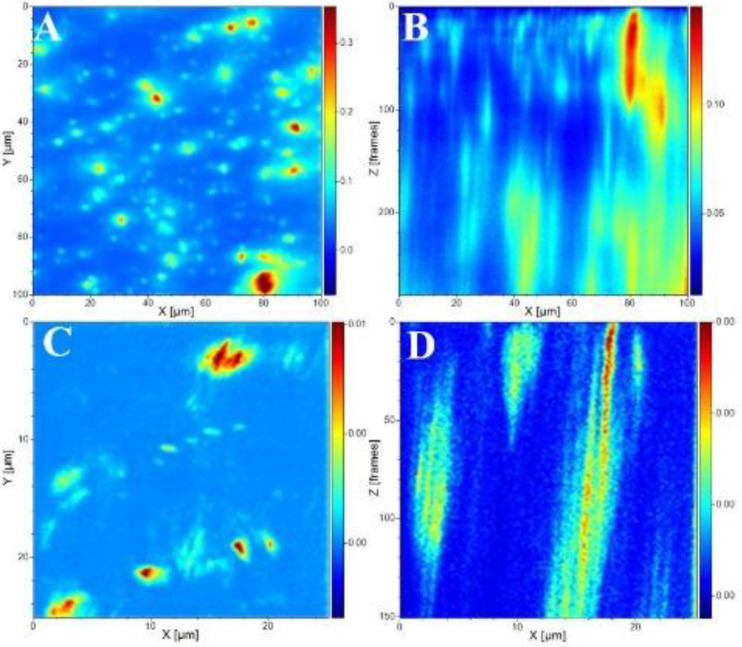
TOF-SIMS image of m/z (Ti element) distribution on (**A**) CM3 membrane sample surface and (**B**) the corresponding depth image, as well as (**C**,**D**) images corresponding to similar measurements along the membrane cross-section.

Figure 4 shows filler distribution maps across the surface (top) and cross-section of the membrane sample CM3. The image reveals that the filler particles are homogeneously distributed throughout the membrane with few aggregations. This is further supported by the depth profile of the Ti element shown in Figure S6.

**Figure 5:**
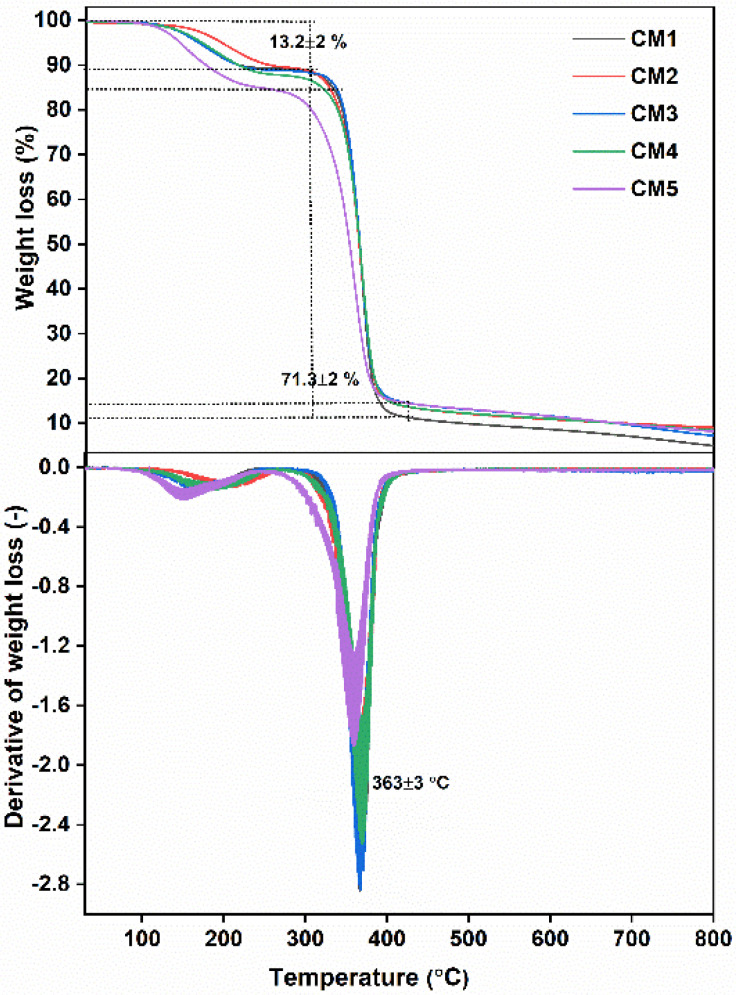
TGA DTG spectra of MMMs with different MXene filler loadings.

The thermal stability of the as-prepared MMMs at various MXene loading concentrations is depicted in Figure 5. TGA and its corresponding DTG spectra revealed two weight loss regions. The first region, at a temperature of 1552013195 °C, is characterized by weight loss of 13.2 ± 2%, which was due to desorption of physically/chemically bound water, removal of trapped solvent, and dissociation of small amounts of esterified chains as well as acetylated cellulose [46]. The second transitional region, at around 363 ± 3 °C, shows a major decomposition range corresponding to a significant weight loss of 85 ± 3%, which appears to be due to degradation of the main polymer chain. The decomposition of MMMs, compared with pristine CTA [47], starts at an elevated temperature due to the strong interactions between the polymer and filler through the polar groups, which decrease the thermal motion of the polymer; thus, the amount of energy required for polymer chain movement or segmentation is increased, leading to the enhanced thermal stability.

**Figure 6:**
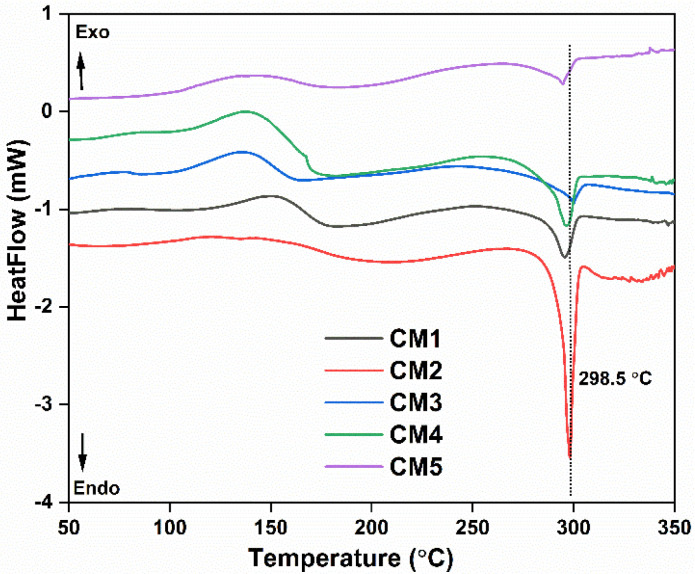
DSC thermogram of the MMMs synthesized with various filler loadings.

Similarly, the thermal behavior of membrane materials was further evaluated through DSC analysis. Figure 6 shows the DSC spectra of synthesized MMMs. The broad exothermic peak centered at 138.2 °C corresponds to the crystallization temperature. Compared with pristine CTA (with a crystallization temperature of 156 °C) [42], the crystallization peak shifted toward the lower temperature side with an increase in filler concentration. Furthermore, the observed broader and lower-intensity peak of the MMMs as compared to pristine CTA is ascribed to the slow crystallization. The addition of MXene fillers possessing polar groups should facilitate coordination with the polymer chains, thus reducing its tendency to form crystalline phases [48]. Similarly, the endothermic peak corresponding to fusion/melting emerges at a temperature of 298.5 °C, higher than for pristine CTA (melting/fusion temperature 289 °C) [47]. These results can be attributed to the excellent interaction and distribution of the filler particles within the polymer matrix.

## 4. Gas Separation Performances Evaluation

**Figure 7:**
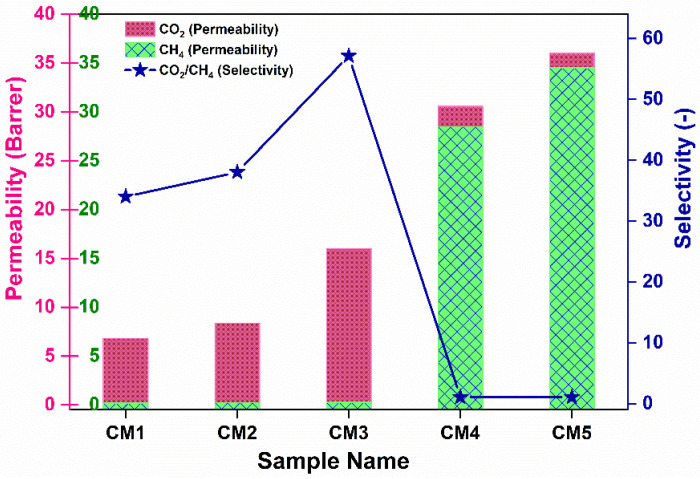
Single gas separation behavior of synthesized MMMs at various concentration of Mxene nanofillers.

To study the CO_2_ and CH_4_ gas transport efficiency of the CTA-based membranes, permeation evaluation was performed in a variable volume-constant pressure custom-built time lag permeation setup. The experiment was operated at 25 °C temperature and 1.5 bar feed pressure. A simple soap film flowmeter was used to obtain the flow rate, which was measured using a soap film flow meter. First, we investigated the effect of MXene concentration on the CO_2_/CH_4_ separation performance. As shown in Figure 7, the pure gas (CO_2_ and CH_4_) gradually increased with MXene loading up to 5 wt.%, with the CO_2_ as well as CH_4_ permeance increasing. Compared to the increase in CH_4_ permeance up to 3 wt.%, a dramatic increase in CH_4_ permeance was observed at higher MXene loadings. Hence, the CO_2_/CH_4_ selectivity increases up to 3 wt.% (reaching 57.14) and then decreases to 5 wt.%. Compared with the CO_2_ permeance of pristine CTA (3.01 Barrer) [47], the permeability of the 3 wt.% MXene-containing CTA sample is increased 5-fold, reaching a permeability of 16 Barrer, with a 2-fold increase in CO_2_/CH_4_ selectivity. Thus, introducing MXene nanosheets (up to 3 wt.%) provides additional molecular transport channels, enhancing CO_2_ permeance and CO_2_/CH_4_ selectivity. The enhancement in gas separation performance is also attributed to the high CO_2_ absorption capacity (Figure S7), due to the presence of abundant polar groups on the MXene surface as well as the assembly of the laminar structure with nanochannels that enhance the diffusion of gas molecules (Figure S8), thus increasing the permeability. Hence, it can be concluded that a solution–diffusion transport mechanism was obeyed. The further decrease in selectivity at a higher loading of MXene above 3 wt.% can be attributed to the generation of non-selective regions due to the accumulation of filler nanoparticles [23].

**Figure 8:**
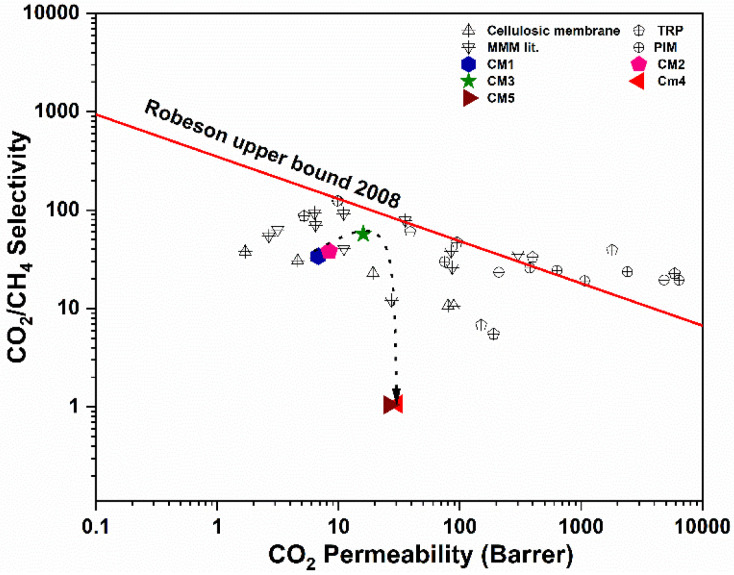
Comparison of CO_2_/CH_4_ separation performance of the as-synthesized membranes, possessing different concentration of MXene filler, with the standard Robeson’s 2008 upper bound plot [49,50]. The dashed arrow serves to guide the eye. Some of the values in the data point are adapted with permission from ref. [49].

Figure 8 compares the CO_2_/CH_4_ permeation efficiency of the synthesized MMMs in this work with the standard Robeson’s 2008 upper bound plot. It is observed that, with an increase in the concentration of MXene as a nanofiller, the permeance and selectivity increase up to 3 wt.%, almost reaching the upper bound, before decreasing in selectivity as the concentration is further increased. The increase in selectivity can be attributed to the high affinity of the fillers for CO_2_.

To further evaluate the drastic change in the permeability and selectivity trend of the MMMs at MXene concentrations higher than 3 wt.%, we measured the permeability of different hydrocarbons such as propane (C_3_H_8_), propylene (C_3_H_6_), butane (C_4_H_10_), and sulfur hexafluoride (SF_6_) through the CM_4_ and CM_5_ membranes. The results are provided in Figure S9. The order of gas permeability for both membranes is P_CH4_ > P_C3H8_ > P_C3H6_ > P_C4H10_ > P_SF6_, which corresponds to the kinetic diameters of the studied gases. In principle, the permeation of gas molecules in polymeric membranes occurs by a solution–diffusion process. However, for membranes with a higher concentration of MXene as a filler (above 4 wt.%), gas separation efficiency is controlled by a diffusion mechanism, which can be ascribed as molecular sieving. Generally, for small and almost spherical gas molecules, the logarithm of diffusion coefficient (*D*) is proportional to the square of the molecular kinetic diameter (*d*), as shown in the equation [51]:

(5) $$log\left(D\right)\propto d^{2}$$

**Figure 9:**
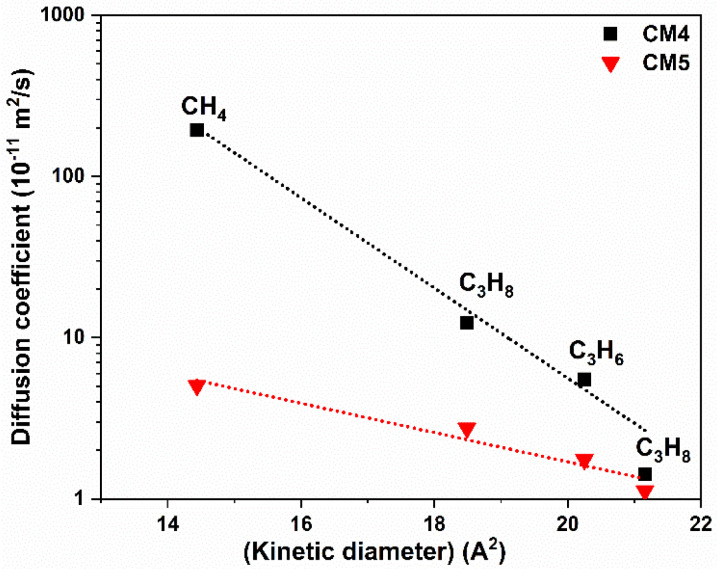
Relation between the diffusion coefficient and the corresponding kinetic diameter of the different hydrocarbons.

Figure 9 shows the relation of log (*D*) and *d*^2^ for the different hydrocarbons. It is observed that this correlation, which states that the diffusion coefficient decreases with the molecular size, is obeyed by the gases in the study.

**Figure 10:**
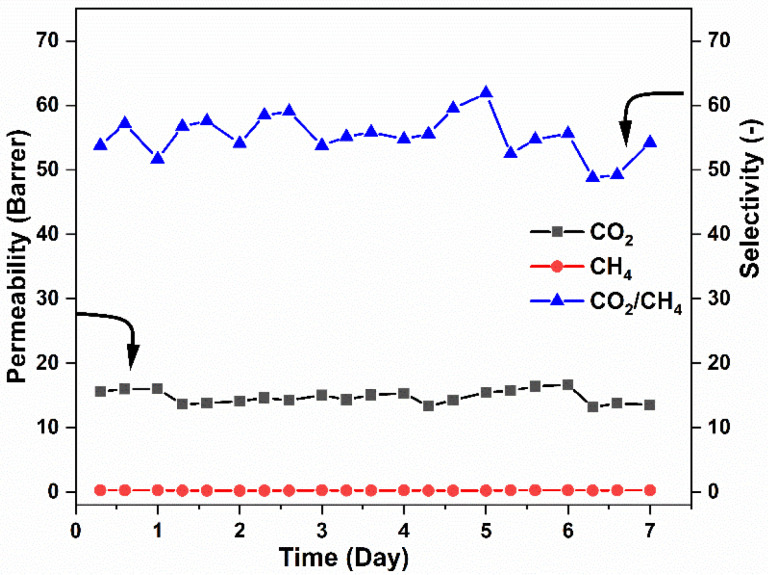
Long-term stability test on CM3 sample for CO_2_ and CH_4_ single-gas separation.

Additionally, the CO_2_/CH_4_ separation efficiency of MXene-CTA MMMs was studied through a one-week-long separation experiment at 1.5 bar pressure and 25 °C temperature. At least three consecutive measurements were performed each day. The deviation in the permeability was calculated to be below 10%. As shown in Figure 10, the permeability and selectivity of the membrane sample (CM3) for CO_2_/CH_4_ separation remained nearly the same at the beginning as after the one-week experiment. This result thus suggests the high stability of the synthesized MMM samples and their practical potential in separation of CO_2_ from bio/natural gas.

To analyze the impact of the filler shape on the gas separation performance, membranes with different fillers—namely, 0D (TiO_2_ nanosphere) and 1D (TiO_2_ nanotube) fillers—were synthesized, and their gas (CO_2_ and CH_4_) separation efficiency was compared with that of a membrane containing 2D (MXene nanosheet) filler.

**Figure 11:**
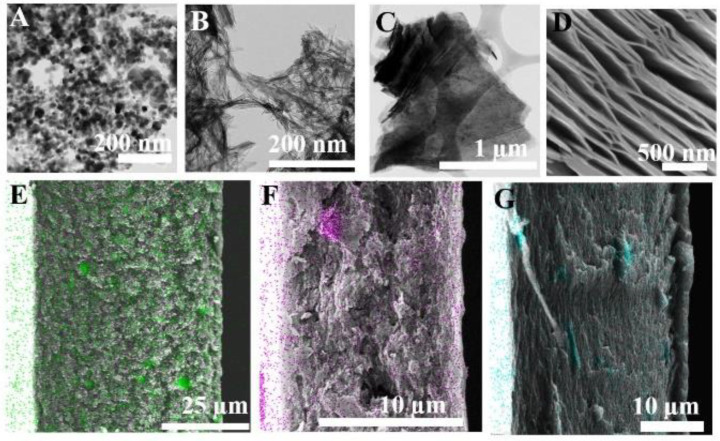
TEM images (**A**–**C**) of the 0D, 1D, and 2D nanofillers; SEM image (**D**) of the 2D nanofiller; and SEM-EDS images of cross-sections of (**E**) 0D, (**F**) 1D, and (**G**) 2D filler-incorporated MMMs overlaid with the distributed corresponding Ti element (colored).

TEM, as well as SEM, was used to evaluate the morphology of the as-synthesized fillers (Figure 11). The TEM image in Figure 11A shows uniform TiO_2_ nanospheres (0D) with an average particle size of 20 nm. Figure 11B demonstrates the thin fiber-like hollow tubular structure of the TiO_2_ nanotubes (1D). Figure 11C, D show the TEM and SEM images of MXene sheets (2D), with large lateral dimensions and rigid sheets that are stacked in an orderly manner, naturally generating interlayer channels through direct self-stacking. Figure 11E–G display the membrane morphology and pattern of the fillers distributed across the membrane. The filler particles were homogeneously distributed throughout the membrane with only a few aggregations, as revealed through elemental mapping. Different membrane morphologies can be obtained by adding fillers of different shapes. Upon addition of 0D and 1D fillers, voids ranging from nm- to µm-scale can be observed, thus improving gas permeability. In contrast, in the case of 2D fillers, a highly compact dense morphology free of voids is observed.

**Figure 12:**
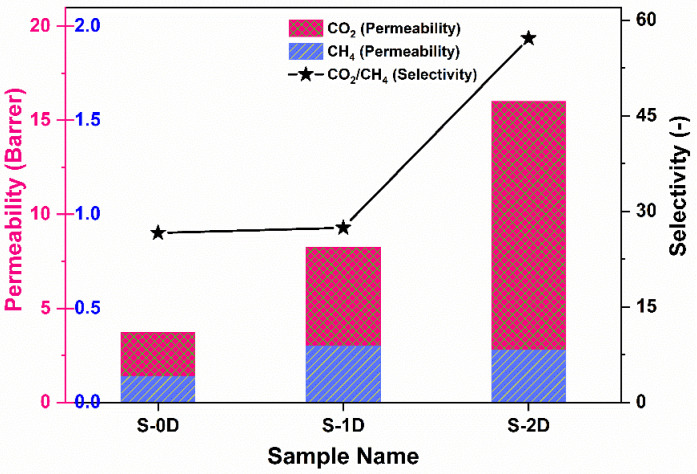
Comparison of gas separation efficiency of MMMs synthesized using 0D, 1D, and 2D fillers.

The efficiencies of different filler loaded MMMs towards gas permeability and selectivity are presented in Figure 12. The CO_2_ permeability and CO_2_/CH_4_ selectivity correspond to the following order: 2D >1D > 0D. The use of 0D fillers (S-0D) marginally increases the permeability of CO_2_ and CO_2_/CH_4_ selectivity compared with pristine CTA polymer, as has been evidenced in our previous work [47]. Such increased selectivity is attributed to the availability of hydroxyl groups on the TiO_2_ surface that have stronger interactions with polar CO_2_ molecules as compared to non-polar CH_4_ molecules, thus promoting increased selectivity. For sample S-1D, permeability of both the CO_2_ and CH_4_ gases increased, while a marginal increase in CO_2_/CH_4_ selectivity was observed. The hollow structure of vertically aligned TNTs can serve as rapid transport channels that function as less-restricted gas diffusional pathways with minimal permeation barrier, thus increasing the permeability of CO_2_ and CH_4_ [33]. However, partial blockage of the tubes by polymer chains is inevitable. It is speculated that embedding 1D fillers provides a tortuous network of diffusion paths for the large molecules such as CH_4_ through the membrane. In the meantime, such a network might not be adequate to moderate the diffusion of smaller molecules, such as CO_2_. Likewise, due to the insufficient interaction between TNT fillers and polymer phase, undesirable channels might be formed at the interfacial region between polymer and fillers, further enabling the transport of CH_4_. The enhanced gas selectivity of membrane S-2D containing 2D filler was ascribed to the preferential horizontal orientation of the sheets, which hinders the transport of larger gas molecules (CH_4_). Similarly, the functional polar groups on the surface can form partial bonds with CO_2_, thus providing a facilitated transport mechanism. Apart from the significant concerns of nanoparticle structures, nanoparticle shapes cannot be avoided when matrix–filler interactions are considered a significant drawback in polymer nanocomposites. The adjustment of nanofiller shapes will automatically lead to variations in the contact areas between polymer matrices and fillers, which effectively determines the volume of the interfacial regions [52] and, thus, ultimately affects gas permeability and selectivity. Further research utilizing molecular simulation is essential, which is expected to further extend knowledge in this area.

## 5. Conclusions

In summary, we first synthesized a series of CTA-based MMMs with different concentrations of MXene fillers and assessed their performance in CO_2_/CH_4_ separation. The sample containing 3 wt.% of embedded MXene exhibited the best separation performance, with a CO_2_ permeability of 16 barrer and ideal CO_2_/CH_4_ selectivity of 57.14. Among the fillers with different shapes (i.e., 0D, 1D, and 2D), the 2D fillers (MXene) stand out as a promising candidate for the preparation of MMMs due to their superior interfacial compatibility with polymer matrix, as depicted by the dense, compact, and homogeneous morphology free of voids as observed by SEM. Additional advantageous features include the presence of abundant polar groups and the lamellar nanosheet arrangement, which provide an additional nanochannel for gas transport with the two-fold enhancement of CO_2_/CH_4_ separation compared with the pristine CTA membrane. The high thermal performance and long-term operational stability of the designed materials represent a new milestone for MMMs as alternatives for applications in challenging natural gas purification applications. This work thus provides insight into the development of MMMs with high performance in gas separation toward overcoming current bottlenecks in this area.

## Data Availability

Not applicable.

## Supplementary Materials

The following supporting information can be downloaded at: https://www.mdpi.com/article/10.3390/membranes12100917/s1, Figure S1: XPS survey spectrum of MXene nanosheets, Figure S2: FTIR of MXene nanosheets, Figure S3: XRD patterns of synthesized MMMs with different loading concentrations of MXene., Figure S4: Surface topography analysis of the prepared MMMs containing different loading concentrations of MXene filler., Figure S5: Water contact angle evaluation of MMMs synthesized with different MXene loading concentrations., Figure S6: Depth profile analysis of Ti element distribution in CM3 sample; A) Surface and, B) cross-section., Figure S7: CO_2_ uptake comparison between pristine polymer and MMM with MXene filler., Figure S8: Diffusion coefficient as a function of MXene fillers concentration., and Figure S9: Permeability comparison of different hydrocarbon and SF6 via CM4 and CM3 membranes.
