# Supplementary material for: Cellulose Triacetate-Based Mixed-Matrix Membranes with MXene 2D Filler—CO2/CH4 Separation Performance and Comparison with TiO2-Based 1D and 0D Fillers
Source: Membranes (Basel). 2022 Sep 22;12(10):917. doi: 10.3390/membranes12100917 (PMC9610833; doi:10.3390/membranes12100917)
Supplement: Supplementary file 1 [file membranes-12-00917-s001.zip › membranes-1890291-supplementary.pdf]

Supplementary Material

Cellulose Triacetate-Based Mixed-Matrix Membranes with MXene 2D Filler—CO_2/CH_4 Separation Performance and Comparison with TiO_2-Based 1D and 0D Fillers

Chhabilal Regmi [1,2,*], Jalal Azadmanjiri [3], Vipin Mishra [4], Zdeněk Sofer [3], Saeed Ashtiani [1] and Karel Friess [1,*]

1 Department of Physical Chemistry, University of Chemistry and Technology, Technická 5, 16628 Prague 6 -Dejvicka, Czech Republic
2 Department of Chemical Engineering, University of Arkansas, Fayetteville, AR 72701, USA
3 Department of Inorganic Chemistry, University of Chemistry and Technology, Technická 5, 16628 Prague 6 - Dejvicka, Czech Republic
4 Department of Glass and Ceramics, University of Chemistry and Technology, Technická 5,

16628 Prague 6 -Dejvicka, Czech Republic

Results and Discussions

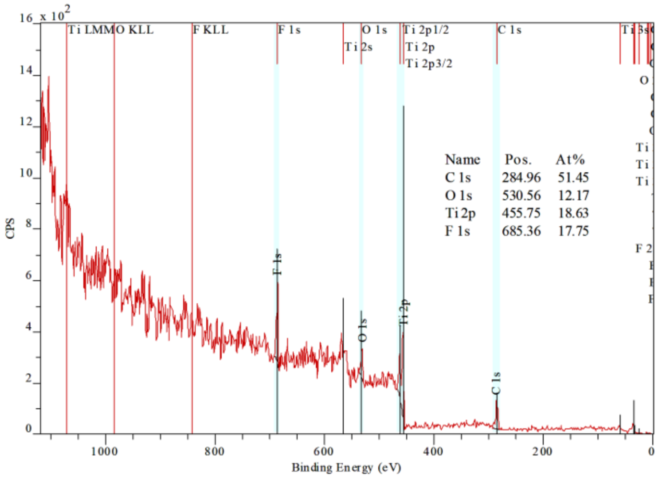

Figure S1 XPS survey spectrum of MXene nanosheets.

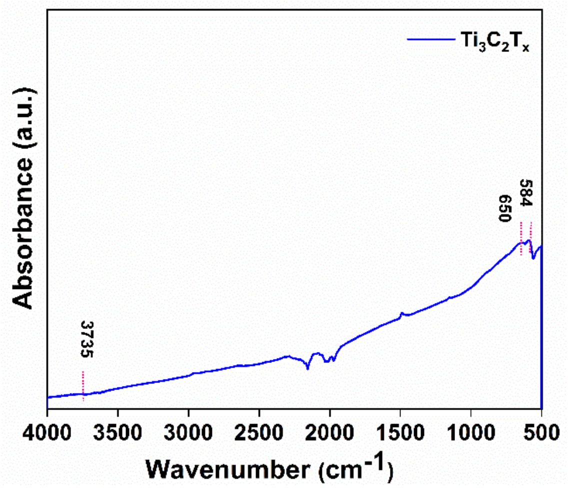

Figure S2 FTIR spectra of MXene nanosheets.

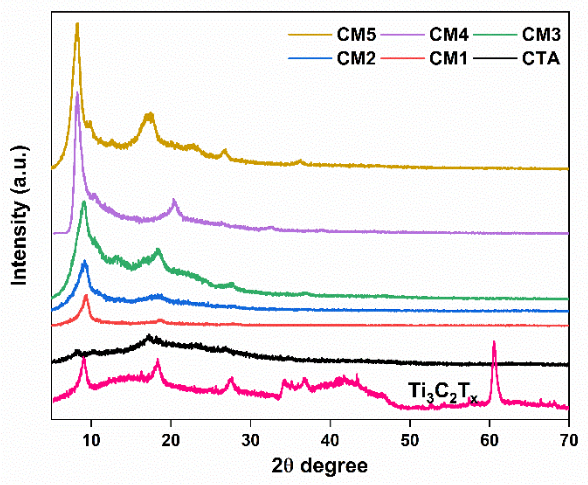

Figure S3 XRD patterns of synthesized MMMs with different loading concentrations of MXene.

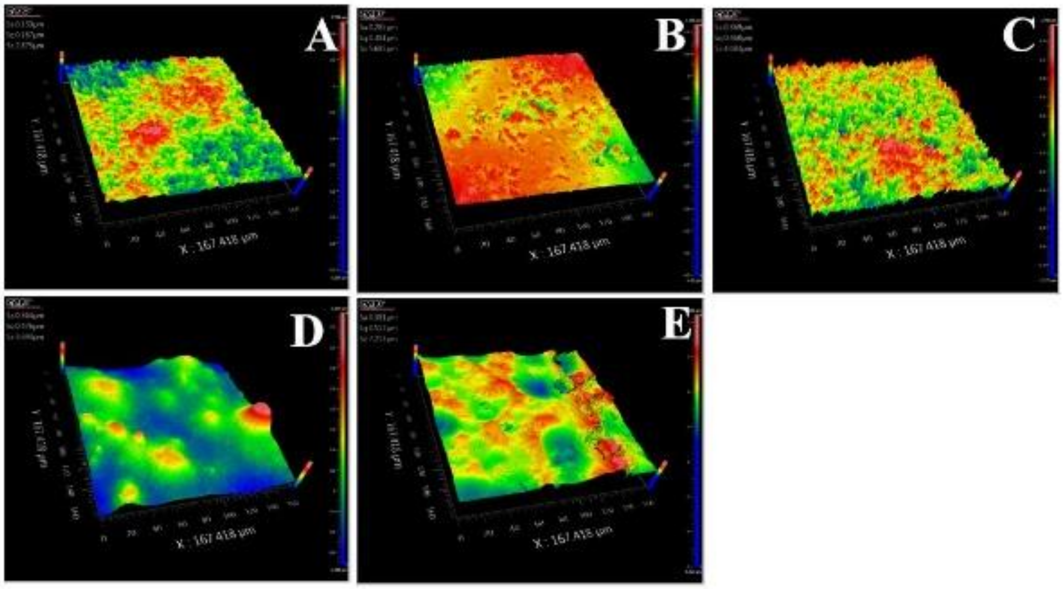

Figure S4 Surface topography analysis of the prepared MMMs containing different loading concentrations of MXene filler.

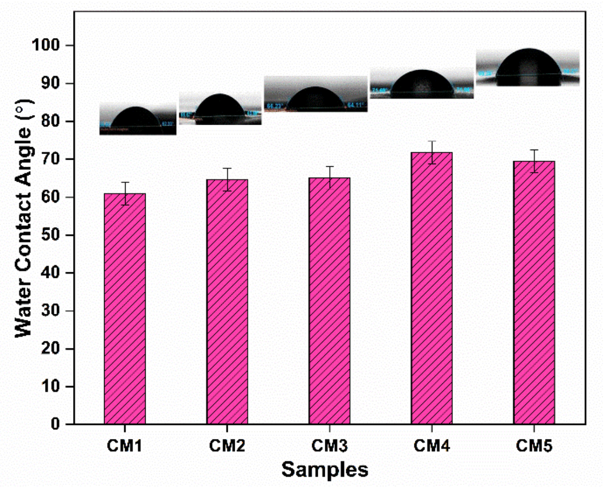

Figure S5 Water contact angle evaluation of MMMs synthesized with different MXene loading concentrations.

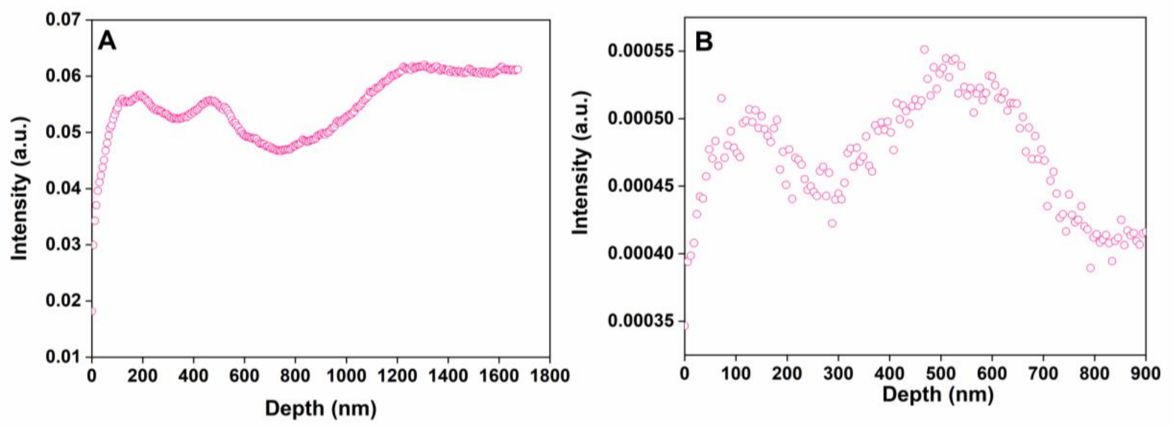

Figure S6 Depth profile analysis of Ti element distribution in CM3 sample A) surface and B) cross-section.

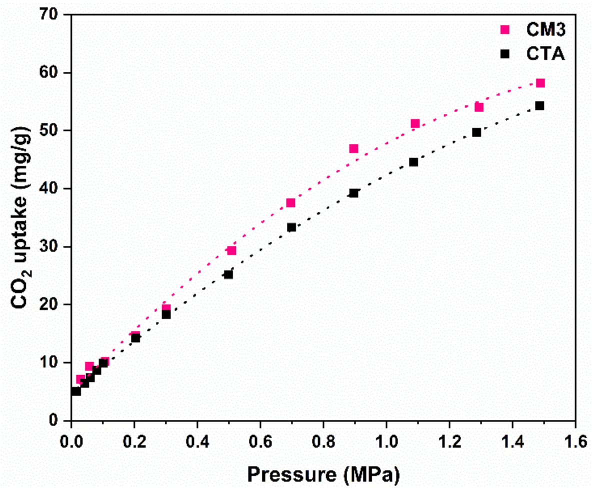

Figure S7 CO_2 uptake comparison between pristine polymer and MMM with MXene filler.

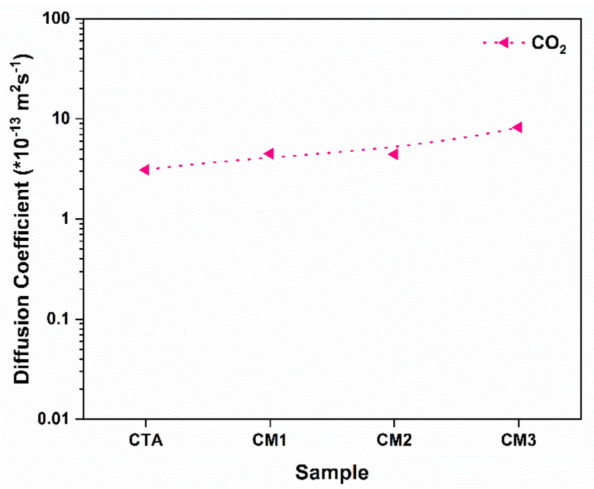

Figure S8 Diffusion coefficient as a function of MXene fillers concentration.

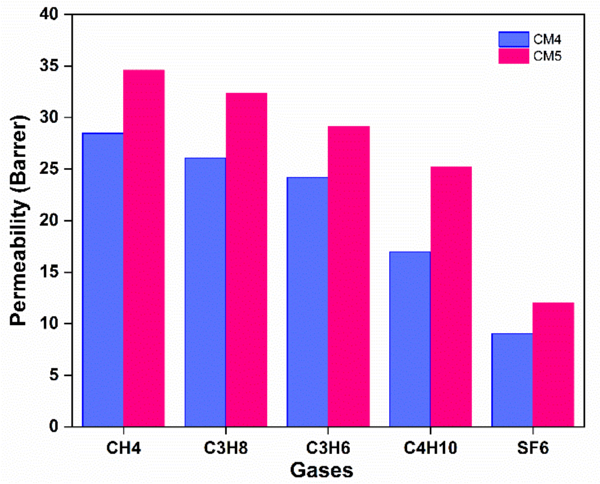

Figure S9 Permeability comparison of different hydrocarbon and SF6 via CM4 and CM5 membranes.
